# Delayed surgery after hip fracture affects the incidence of venous thromboembolism

**DOI:** 10.1186/s13018-023-04122-8

**Published:** 2023-08-28

**Authors:** Takuya Taoka, Takao Ohmori, Tomoko Kanazawa, Kazukiyo Toda, Takeshi Ishihara, Yasuo Ito

**Affiliations:** Department of Orthopaedic Surgery, Kobe Red Cross Hospital, Kobe City, Hyogo 651-0073 Japan

**Keywords:** Hip fracture, Deep venous thrombosis, Pulmonary embolism, Venous thromboembolism, Trauma

## Abstract

**Background:**

Venous thromboembolism (VTE) is one of the most common complications of hip fracture surgeries, and it is unclear whether delayed surgery affects the incidence of VTE. This study aimed to examine the association between delayed surgery and VTE incidence by statistically adjusting for factors that may influence VTE incidence.

**Methods:**

We included 862 patients ≥ 65 years with hip fractures who underwent surgery between October 2010 and December 2020. We examined the effect of surgical delay 48 h after injury on postoperative VTE. Patients with and without VTE were assigned to groups V and NV, respectively. Those with and without proximal deep venous thrombosis (DVT) were assigned to PD and NPD groups, respectively. Univariate analysis was performed to identify factors that might influence DVT development. Risk factors for developing VTE and proximal DVT were analyzed using logistic regression analysis to determine whether delayed surgery was a risk factor.

**Results:**

VTE was observed in 436 patients (40%) and proximal DVT in 48 patients (5.6%). Univariate analysis showed significant differences in the time from trauma to surgery between the V and NV groups and between the PD and NPD groups. In multivariate analysis, surgery 48 h later was also a risk factor for developing VTE and proximal DVT.

**Conclusion:**

A delay in surgery beyond 48 h after a hip fracture injury is a risk factor for developing VTE and proximal DVT.

## Background

Hip fractures are common in older adults [1]. With increasing life expectancy, the incidence of hip fractures is increasing [2]. Therefore, it is important to not only treat fractures but also prevent patient complications [3]. Venous thromboembolism (VTE) is one of the most common complications of hip fracture surgery, and pulmonary embolism (PE) is the fourth leading cause of death in patients with hip fracture [4]. Deep venous thrombosis (DVT) is a major cause of PE; hence its prevention is very important [5]. Reports examining complications after hip fracture surgery found no association between the occurrence of DVT and delayed surgery [6, 7]. However, these studies were unclear on how to assess DVT, and the incidence of DVT was very low, ranging from 0.8 to 1.4%.

Recent studies have, however, reported that the incidence of DVT ranges from 11 to 57% [8–11]. The incidence of DVT from these studies differs significantly from that in studies denying the association.

DVT is often asymptomatic [12] and can occur outside the injured limb [13]. Studies reporting no association may have inaccurately assessed the occurrence of VTE [6, 7]. Additionally, many factors [14] influence the development of VTE, and it is unclear whether delayed surgery influences VTE incidence.

This study aimed to examine the association between delayed surgery and the incidence of VTE by statistically adjusting for factors that influence the incidence of VTE.

## Methods

### Study design

This single-center retrospective cohort study was conducted based on Strengthening the Reporting of Observational Studies in Epidemiology statement.

Our study was approved by the institution’s ethics committee, and the requirement for informed consent was waived due to the retrospective nature of the study.

We included 1119 cases of hip fractures treated between October 2010 and December 2020. The exclusion criteria were as follows: (1) patients ≤ 65 years of age; (2) no postoperative duplex ultrasonography; (3) no body mass index (BMI) data; and (4) simultaneous bilateral hip fractures.

### Protocol for the prevention of VTE in hip fracture

Preoperative: Patients were encouraged to wear elastic stockings on both lower limbs. Automatic ankle joint movement was encouraged to the maximum extent possible.

Intraoperative: Intermittent pneumatic compression device worn on the healthy side.

Postoperative: Intermittent pneumatic compression device worn on both sides.

Patients without contraindications were treated with anticoagulants. On postoperative day 5, DVT of both the lower extremities was evaluated using duplex ultrasonography.

### DVT definition and data collection

Distal DVT was defined as a thrombus in the tibial, peroneal, soleus, or gastrocnemius vein. Proximal DVT was defined as a thrombus in the popliteal vein or a vein proximal to the popliteal vein [15]. If distal and proximal DVT occurred together, we classified it as proximal DVT [16]. Based on medical records, we examined factors that might influence the development of DVT. Factors influencing DVT were age at injury, sex, BMI, fracture type, operative technique, time from injury to surgery, time from surgery to echo, and postoperative anticoagulation therapy, based on previous reports [17].

It has been reported that surgery within 48 h of admission improves outcomes [17]. However, there are cases where this time elapses between injury and hospitalization. We examined patients who underwent surgery within 48 h of injury and those who underwent surgery after this duration.

Patients with and without VTE were classified into the V and NV groups, respectively. Patients with and without proximal DVT were classified into the PD and NPD groups, respectively.

### Statistical analysis

Factors that may influence the development of DVT were compared in univariate and multivariate analyses between the V and NV groups and between the PD and NPD groups.

In univariate analysis, the Mann–Whitney test was used for numerical data and Fisher's exact test for categorical data. Additionally, the risk factors for the development of VTE and proximal DVT were analyzed using logistic regression analysis to test whether the duration of the surgical waiting period was a risk factor.

All *p* values were two-sided, and results were considered statistically significant at *p* values < 0.05. All statistical analyses were performed using EZR (Saitama Medical Center, Jichi Medical University, Saitama, Japan), a graphical user interface for R (The R Foundation for Statistical Computing, Vienna, Austria). It is a modified version of the R software focusing on statistical functions frequently used in biostatistics.

## Results

**Fig. 1:**
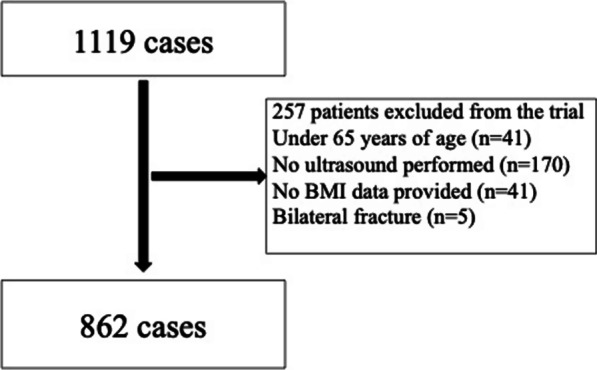
Patient selection flowchart. Overall, 41 cases of patients under 65 years old, 170 cases of no ultrasound performed, 41 cases of no data description of BMI, and five cases of bilateral fracture were excluded. BMI, body mass index

The criteria were met in 862 cases (Fig. 1). VTE was observed in 436 patients (40%), proximal DVT in 48 patients (5.6%), and asymptomatic PE in two patients (0.2%). Symptomatic PE was not observed.

### Univariate analysis

Univariate analysis between the V and NV groups revealed significant differences in age, sex, surgical technique, and waiting period for surgery (*p* < 0.01, *p* < 0.01, *p* < 0.05, *p* < 0.01) (Table 1). There were also significant differences between the PD and NPD groups in terms of BMI and the waiting period for surgery (*p* < 0.01) (Table 2).

**Table 1:** Comparison between VTE and non-VTE groups

	Group V (n = 346)	Group NV (n = 516)	*p* value
Age (years)	86.0	83.0	*p* < 0.01
Sex			*p* < 0.01
Male	53 (15%)	130 (25%)	
Female	293 (85%)	386 (75%)	
BMI (kg/m^2^)	20.2	20.4	0.35
Fracture type			0.60
Femoral neck	186 (54%)	266 (52%)	
Intertrochanteric	151(44%)	240 (47%)	
Subtrochanteric	9 (2%)	10 (1%)	
Operationtechnique			*p* < 0.05
ORIF	226 (65%)	371 (72%)	
Arthroplasty	120 (35%)	145 (28%)	
Time from trauma to surgery			*p* < 0.01
< 48 h	167 (48%)	308 (60%)	
≥ 48 h	179 (52%)	208 (40%)	
Time from surgery to ultrasound (day)	5.0	5.0	0.31
Postoperative anticoagulant			0.61
Nothing	111 (32%)	150 (29%)	
Edoxaban	150 (43%)	214 (41%)	
Enoxaparin sodium	9 (2%)	16 (3%)	
Fondaparinux sodium	76 (22%)	109 (21%)	

*ORIF* Open Reduction and Internal Fixation, *BMI* body mass index, *DVT* deep venous thrombosis, *V* patients with venous thromboembolism, *NV* patients without venous thromboembolism

**Table 2:** Comparison between proximal and non-proximal DVT groups

	Group PD (n = 48)	Group NPD (n = 814)	*p* value
Age (years)	85.5	84.0	0.16
Sex			0.20
Male	14 (29%)	169 (21%)	
Female	34 (71%)	645 (79%)	
BMI (kg/m^2^)	19.0	20.4	*p* < 0.01
Fracture type			0.71
Femoral neck	23 (48%)	429 (53%)	
Intertrochanteric	24 (50%)	367 (45%)	
Subtrochanteric	1 (2%)	18 (2%)	
Operation technique			0.63
ORIF	35 (73%)	562 (69%)	
Arthroplasty	13 (27%)	252 (31%)	
Time from trauma to surgery (h)			*p* < 0.01
< 48 h	19 (40%)	456 (56%)	
≥ 48 h	29 (60%)	358 (44%)	
Time from surgery to ultrasound (day)	6.0	5.0	0.12
Postoperative anticoagulant			0.7
Nothing	17 (35%)	244 (30%)	
Edoxaban	20 (42%)	371 (46%)	
Enoxaparin sodium	2 (4%)	23 (2%)	
Fondaparinux sodium	9 (18%)	176 (22%)	

*ORIF* Open Reduction and Internal Fixation, *BMI* body mass index, *DVT* deep venous thrombosis, *PD* patients with proximal DVT, *NPD* patients without proximal DVT

### Multivariate analysis

Multivariate analysis revealed that age, female sex, and surgery 48 h later (odds ratio [OR] = 1.04, 95% confidence interval [CI]: 1.02–1.06, *p* < 0.01; OR = 1.76, 95% CI 1.22–2.53, *p* < 0.01, and OR = 1.83, 95% CI 1.21–2.20, *p* < 0.01, respectively) were risk factors for VTE (Table 3). BMI and surgery 48 h later (OR = 0.90, 95% CI 0.82–0.99, *p* < 0.05 and OR = 2.17, 95% CI 1.15–4.08, *p* < 0.05, respectively) were risk factors for developing proximal DVT (Table 4).

**Table 3:** Risk factors for postoperative VTE using logistic regression analysis

	Odds ratio	95% CI	*p* value
Age (years)	1.04	1.02–1.06	< 0.01
Sex			
Male (ref.)			
Female	1.76	1.22–2.53	< 0.01
BMI (kg/m^2^)	0.99	0.96–1.04	0.84
Fracture type			
Femoral neck (ref.)			
Intertrochanteric	1.03	0.71–1.5	0.86
Subtrochanteric	1.56	0.59–4.13	0.37
Operation technique			
ORIF (ref.)			
Arthroplasty	1.26	0.84–1.80	0.26
Time from trauma to surgery (h)			
Group E (ref.)			
Group L	1.63	1.21–2.20	< 0.01
Time from surgery to ultrasound (day)	1.02	0.98–1.07	0.35
Postoperative anticoagulant			
Nothing (ref.)			
Edoxaban	1.01	0.72–1.41	0.97
Enoxaparin sodium	0.73	0.29–1.70	0.43
Fondaparinux sodium	1.1	0.73–2.20	0.64

*ORIF* Open Reduction and Internal Fixation, *BMI* body mass index, *CI* confidence interval, *ref* reference, *VTE* venous thromboembolism

**Table 4:** Risk factors for postoperative proximal DVT

	Odds ratio	95% CI	*p* value
Age (years)	1.03	0.98, 1.07	0.24
Sex			
Male (ref.)			
Female	0.61	0.31, 1.18	0.14
BMI (kg/m^2^)	0.9	0.82, 0.99	< 0.05
Fracture type			
Femoral neck (ref.)			
Intertrochanteric	1.1	0.51, 2.39	0.8
Subtrochanteric	0.96	0.11, 8.22	0.97
Operation technique			
ORIF (ref.)			
Arthroplasty	0.76	0.32, 1.18	0.55
Time from trauma to surgery (h)			
Group E (ref.)			
Group L	2.17	1.15, 4.08	< 0.05
Time from surgery to ultrasound (day)	1.06	0.98, 1.16	0.16
Postoperative anticoagulant			
Nothing			
Edoxaban	0.89	0.44, 1.78	0.74
Enoxaparin sodium	1.13	0.24, 5.38	0.88
Fondaparinux sodium	0.88	0.37, 2.12	0.79

*ORIF* Open Reduction and Internal Fixation, *BMI* body mass index, *CI* confidence interval, *ref* reference, *DVT* deep venous thrombosis

## Discussion

In this study, we screened for DVT using lower-extremity duplex ultrasonography after hip fracture surgery and statistically adjusted for factors that may influence the development of VTE, including the time from trauma to surgery.

The incidence of postoperative DVT was 40%. Delayed surgery is a risk factor for VTE development. Delayed surgery is also a risk factor for proximal DVT, which is associated with a high risk of developing PE.

Some reports with large surveys found no association between delayed surgery and VTE [5, 6]. These studies reported a DVT incidence of 0.8–1.9% and a PE incidence of 0.7–1.7%. Compared with the present study, the incidence of PE is comparable, but the incidence of DVT is very low, and the method of testing for DVT is unknown. Screening tests were not performed, and DVT was likely not diagnosed. Patients with symptomatic VTE present with swelling, pain, erythema, localized heat in the lower extremities, and dyspnea. Swelling and pain are often difficult to diagnose when symptoms overlap with those associated with proximal femoral fractures or asymptomatic VTE [18]. It can occur on the healthy and injured sides 10, and a screening examination of both lower extremities is necessary.

The present study included cases in which screening tests were performed on both lower extremities to diagnose symptomatic and asymptomatic DVT. Zhang et al. performed a multivariate analysis of 463 proximal femoral fractures that underwent ultrasonography at screening and reported that the duration of time waiting for surgery was a risk factor for the development of DVT [8]. However, the study did not distinguish between distal and proximal DVT. Proximal DVT is associated with a higher risk of developing PE than distal DVT [19–21], and its impact on proximal DVT is clinically significant. Therefore, we investigated the relationship between proximal DVT and surgical waiting time. The waiting time for surgery was also a risk factor for proximal DVT.

However, a recent study stated that distal DVT could progress to proximal DVT and PE [22]. It should be noted that distal DVT is associated with the same risk of developing PE as proximal DVT [23]. In the present study, we examined distal and proximal types of VTE and DVT and found that the duration of the delayed surgery was a risk factor for both types of VTE. Surgery within 48 h is important to prevent postoperative VTE.

The limitations of this study include the following:

1. This was a single-center retrospective study, which may have affected the accuracy and precision of data collection and introduced unavoidable selection bias.
2. Although there were reports of preoperative DVT, we performed surgery early in many cases and did not perform a preoperative thrombogenic evaluation.
3. We did not include all factors, such as history of hospitalization, postoperative mobilization, cancer, or presence of infection, involved in DVT.

## Conclusions

Surgery for proximal femur fractures 48 h after an injury is a risk factor for developing VTE and proximal DVT. Surgery within 48 h is important to prevent postoperative VTE.

## Data Availability

The datasets used and/or analyzed during the current study are available from the corresponding author on reasonable request.

## Abbreviations

VTE: Venous thromboembolism
PE: Pulmonary embolism
DVT: Deep venous thrombosis
PD: Patients with proximal DVT
NPD: Patients without proximal DVT
V: Patients with VTE
NV: Patients without VTE
OR: Odds ratio
CI: Confidence interval
